# MacroH2A2-Enriched Domains Are Largely Stable Across the Cell Cycle but Focally Displaced at Mitotic Regulatory Elements

**DOI:** 10.3390/ijms27177708

**Published:** 2026-08-28

**Authors:** Yongzhuo Deng, Zeqian Xu, Le Zhang, Jinling Liu, Chuansheng Hu, Zihan Shi, Xinhui Li, Zhifeng Shao

**Affiliations:** 1 School of Biomedical Engineering, Shanghai Jiao Tong University, Shanghai 200240, China; yzdeng@sjtu.edu.cn (Y.D.); xzq_1995@sjtu.edu.cn (Z.X.); zlehit@sjtu.edu.cn (L.Z.); missliujinling1@sjtu.edu.cn (J.L.); huchuansheng@gmail.com (C.H.); shizihan@sjtu.edu.cn (Z.S.); zfshao@sjtu.edu.cn (Z.S.); 2 National Engineering Research Center of Advanced Magnetic Resonance Technologies for Diagnosis and Therapy (NERC-AMRT), Shanghai Jiao Tong University, Shanghai 200240, China

**Keywords:** histone variant macroH2A2, nChIP-seq, cell cycle, chromatin accessibility, transcriptional regulation

## Abstract

The macroH2A variants mH2A1 and mH2A2 are structurally similar but not identical. Our previous study demonstrated that mH2A1 is reloaded during cell-cycle progression, but whether mH2A2 follows similar dynamics has remained unclear. Here, we used native ChIP-seq in synchronized Huh-7 cells to profile both variants at G1/S and G2/M. Although mH2A1- and mH2A2-enriched domains overlapped extensively, mH2A2 domains were largely stable across the cell cycle, in sharp contrast to the dynamic reloading of mH2A1. Only a small subset of mH2A2 domains showed phase-specific deposition or displacement. Among these, G1/S-unique mH2A2 domains were preferentially located in the active A compartment and coincided with reduced chromatin accessibility at binding sites for cell-cycle regulators. These G1/S-unique domains co-localize with genes involved in mitotic progression within the same A compartment, suggesting potential regulatory roles in both chromatin organization and transcriptional regulation. These findings refine the classical view of macroH2A variants as static repressive marks: mH2A2 is not entirely static, but its cell-cycle dynamics are far more restricted than those of its paralog mH2A1, occurring only at a small subset of genomic loci, with a regulatory logic distinct from that of mH2A1.

## 1. Introduction

The replacement of canonical histones by histone variants provides chromatin with unique regulatory properties and contributes to diverse genomic processes [1,2]. Among these variants, macroH2A (mH2A) is distinguished by a 30 kDa macrodomain at its C-terminus [3]. This mH2A family comprises two proteins encoded by separate genes: mH2A1, encoded by *H2AFY*, and mH2A2, encoded by *H2AFY2* [4].

mH2A variants were first characterized by their enrichment in facultative heterochromatin and on the inactive X chromosome, where they occupy broad domains spanning several kilobases [5]. These domains often cover gene bodies and restrict the access of transcription factors and chromatin-associated complexes to regulatory regions, supporting the established role of mH2A variants as transcriptional repressors [6,7]. Beyond transcriptional repression, mH2A variants also contribute to higher-order chromatin organization, including the anchoring of structural domains to the nuclear lamina [8]. They further act as an epigenetic barrier that helps maintain somatic cell identity and resists chromatin reprogramming towards pluripotency [9]. Together, these findings define mH2A variants as multifunctional regulators of chromatin state and cellular identity.

Although mH2A1 and mH2A2 are structurally related, accumulating evidence indicates that they are not functionally redundant [9,10]. In contrast to mH2A1, which is strongly associated with broad repressive domains at gene bodies, mH2A2 can regulate enhancer accessibility and suppress self-renewal and stemness-associated transcriptional programs by disrupting distal interactions [11]. mH2A2 also contributes to cell-identity maintenance through mechanisms different from those mediated by mH2A1 [10]. In addition, mH2A2 has been implicated as a barrier to epithelial–mesenchymal transition and tumor progression, further highlighting its non-redundant role in limiting cellular plasticity [12].

Cell-cycle progression is accompanied by phase-specific transcriptional and chromatin changes, allowing different sets of genes to be activated or repressed at certain stages [13,14]. Our previous work showed that mH2A1 undergoes cell-cycle-dependent deposition and accumulates on chromatin during mitosis, mainly across gene bodies [15]. Consistent with this dynamic behavior, mH2A1 contributes to replication timing control, as its loss causes asynchronous firing of replication origins on the inactive X chromosome [16]. However, whether mH2A2 follows similar cell-cycle dynamics remains unclear. In addition, it is also unknown whether mH2A2 modulates the accessibility of regulatory elements at certain stages.

To address these questions, we performed native ChIP-seq (nChIP-seq) in synchronized Huh-7 cells and profiled mH2A1- and mH2A2-enriched domains at G1/S and G2/M states. By comparing their genome-wide distribution between these two cell-cycle states, we found that mH2A2 exhibits a regulatory landscape distinct from that of mH2A1. In contrast to the broader dynamics of mH2A1, mH2A2 remains largely stable across the genome but is selectively displaced from a subset of regions during G2/M. Notably, these displaced regions contain a significant number of binding sites for multiple mitotic regulators. These findings raise the possibility that mH2A2 contributes to cell-cycle-dependent regulation of chromatin accessibility at specific regulatory elements.

## 2. Results

### 2.1. mH2A2 Broadly Overlaps with mH2A1-Enriched Domains but Shows Distinct Local Enrichment

To generate high-confidence genomic maps of macroH2A variants, we first accessed cell synchronization efficiency and sequencing alignment quality (Supplementary Figure S1, Supplementary Table S1). All independent biological replicates showed similar mono-nucleosomal fragment-size distributions and high reproducibility, as indicated by strong Pearson correlation coefficients between replicates (Supplementary Figure S2). We therefore merged biological replicates for downstream analyses.

**Figure 1:**
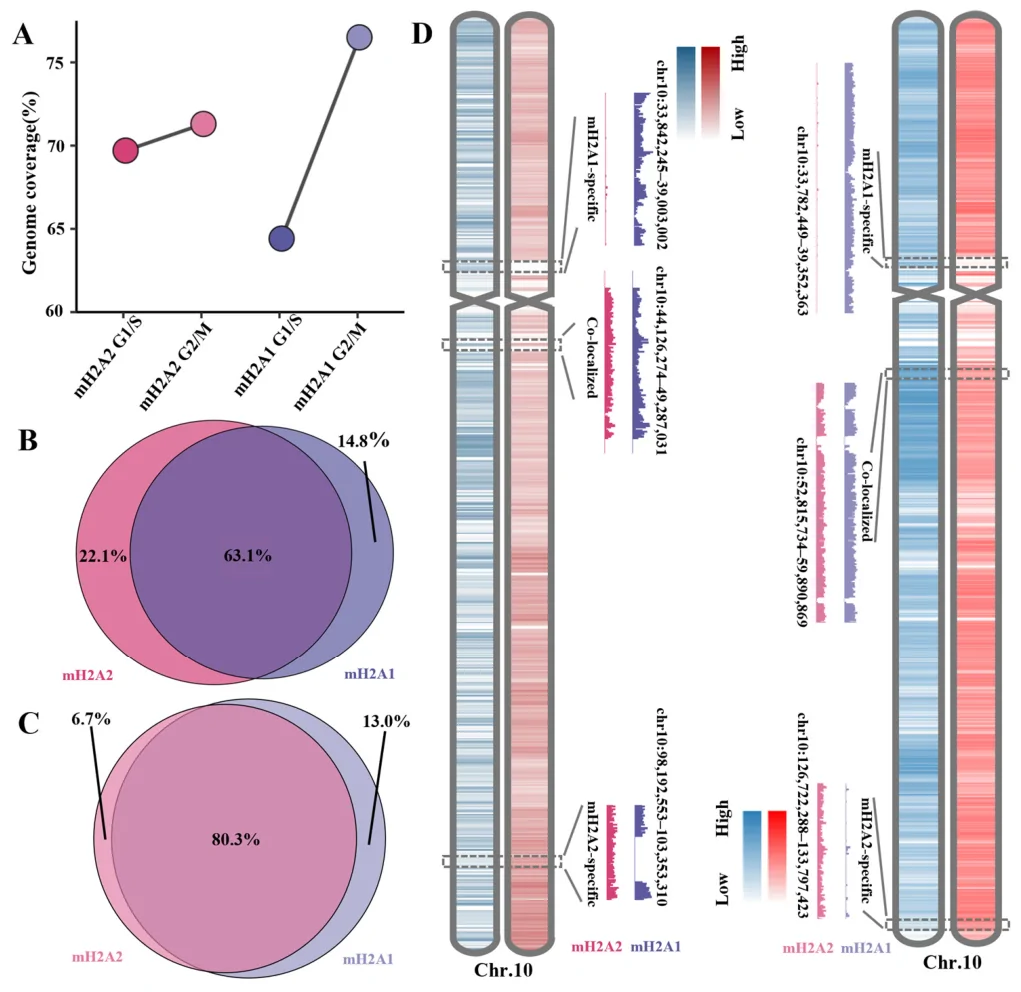
Genome-wide co-occupied and variant-specific mH2A2 and mH2A1 coverage across the cell cycle. (**A**) Genomic coverage (%) of mH2A1 and mH2A2 domains G1/S and G2/M. (**B**,**C**) Overlap between mH2A2 and mH2A1 domains at G1/S (**B**) and G2/M (**C**). (**D**) Chromosome-wide heatmaps of mH2A1 and mH2A2 enrichment on Chr. 10 at G1/S (**left**) and G2/M (**right**), showing representative mH2A1-specific, mH2A2-specific, and co-occupied regions.

Consistent with previous studies, both mH2A1 and mH2A2 nucleosomes formed broad enriched domains across the genome [15,17]. Overall, we identified 36,088 mH2A1-enriched domains at G1/S and 41,691 at G2/M, with median sizes of 24 kb and 30 kb, respectively. These domains covered 63.4% of the genome at G1/S (1966 Mb) and 76.5% of the genome at G2/M (2372 Mb). For mH2A2, we identified 36,742 enriched domains at G1/S and 41,625 at G2/M, with a consistent median size of 22 kb. These domains covered 69.7% of the genome at G1/S (2153 Mb) and 71.3% at G2/M (2212 Mb; Figure 1A).

We next measured the genomic coverage shared by, or specific to, each mH2A variant at G1/S and G2/M. At G1/S, 51.4% of the genome (1593 Mb) was co-occupied by both mH2A1 and mH2A2 domains, whereas 12.0% (373 Mb) and 18.1% (560 Mb) were specifically covered by mH2A1 and mH2A2 domains, respectively (Figure 1B). At G2/M, the co-occupied fraction increased to 65.9% of the genome (2042 Mb), while mH2A1-specific and mH2A2-specific coverage decreased to 10.6% (330 Mb) and 5.5% (169 Mb), respectively (Figure 1C).

Although mH2A1 and mH2A2 domains overlapped extensively, their local enrichment levels differed (Figure 1D). Among co-occupied regions at G1/S, 48.5% showed comparable enrichment of the two variants, whereas 43.8% showed higher mH2A1 enrichment and 7.7% showed higher mH2A2 enrichment (Supplementary Figure S4).

### 2.2. mH2A2 Localizes to Gene Bodies Without Uniformly Silencing Transcription

**Figure 2:**
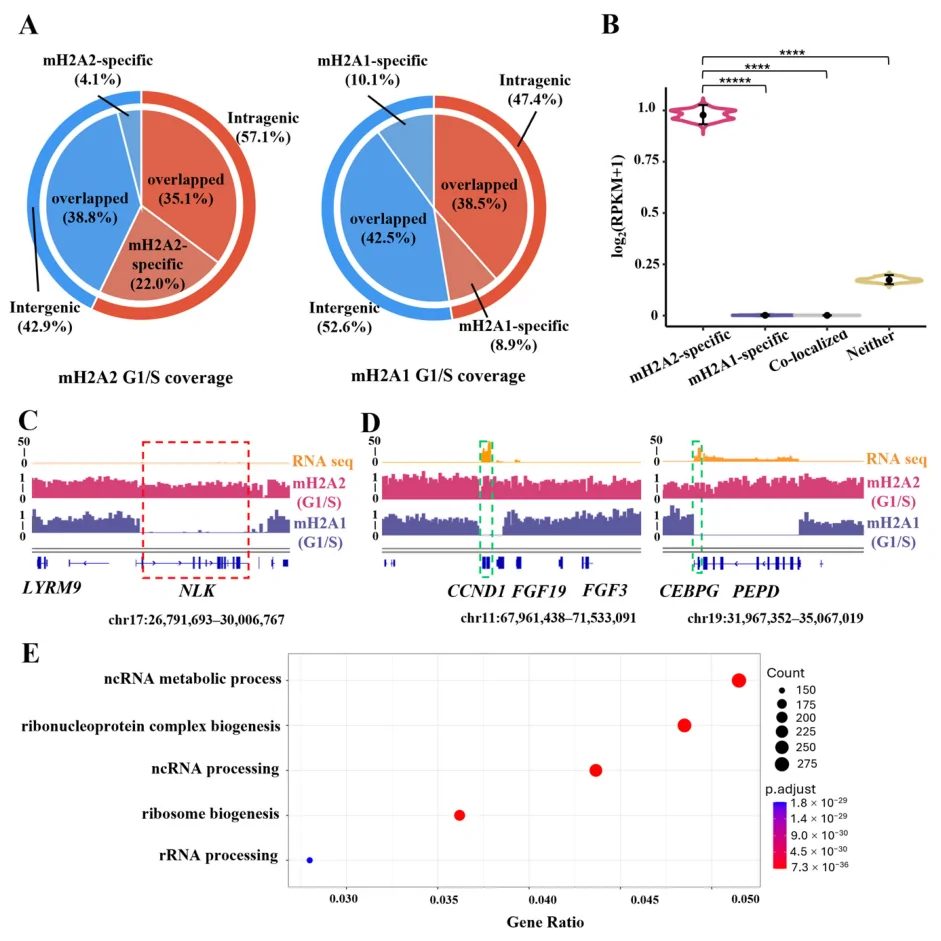
Genomic distribution and transcriptional states of genes covered by mH2A variants at G1/S. (**A**) Proportions of co-occupied and variant-specific genomic coverage. (**B**) Violin plots of gene expression levels (RPKM) for mH2A2-specific, mH2A1-specific, co-localized, and uncovered genes. Medians are marked within each violin. Statistical significance was evaluated by two-tailed non-parametric Wilcoxon rank-sum tests and adjusted by the Benjamini–Hochberg (BH) method (****, adjusted *p*-value < 0.0001; *****, adjusted *p*-value < 0.00001). (**C**,**D**) IGV views of RNA-seq, mH2A2, and mH2A1 signals at representative lowly expressed (**C**) and active (**D**) mH2A2-covered genes; RPKM values: *NLK* 0.14, *CCND1* 36.5, *CEBPG* 33.9. The red and green dashed squares mark genes with low and high expression levels, respectively. (**E**) GO enrichment of actively transcribed mH2A2-covered genes (RPKM > 1).

Because both mH2A variants are implicated in transcriptional regulation, we first examined their distribution across intergenic and intragenic regions at G1/S (Figure 2A). Regions co-occupied by mH2A1 and mH2A2 were distributed comparably between intergenic (27.0% of the genome) and intragenic (24.4%) regions. mH2A1-specific domains showed a similarly even distribution, occupying 6.4% of the genome in intergenic regions and 5.6% in intragenic regions. By contrast, mH2A2-specific domains were strongly biased toward gene bodies (15.3% of the genome), with only a small fraction (2.8% of the genome) in intergenic regions.

We next compared gene expression according to mH2A occupancy status at G1/S. Based on their overlap with mH2A variant domains, genes were grouped as mH2A1-specific, mH2A2-specific, co-localized or uncovered by either variant. Consistent with the established role of mH2A1 in transcriptional repression, mH2A1-specific and co-localized genes were largely suppressed (Figure 2B). Genes lacking either variant showed detectable but generally low expression levels. In contrast, mH2A2-specific genes spanned a broad transcriptional spectrum, ranging from unexpressed (RPKM = 0; 948), through lowly expressed (0 < RPKM < 1; 2005), to highly expressed (RPKM > 1; 2899) genes. For example, *NLK*, which mediates SOX11-induced growth inhibition in hepatocellular carcinoma [18], was lowly expressed (RPKM = 0.14) despite mH2A2 occupancy across its gene body. By contrast, cell-cycle- and proliferation-related genes such as *CCND1* and *CEBPG* retained clear expression while being occupied by mH2A2 domains (Figure 2C,D) [19,20]. To determine whether the expressed mH2A2-specific genes were functionally related, we performed GO analysis on mH2A2-specific transcribed genes (RPKM > 1) [21]. These genes were enriched for pathways related to ncRNA metabolism and ribosome biogenesis (Figure 2E). To visualize the relationship between mH2A variant occupancy and gene expression, we generated a coverage heatmap of mH2A1 and mH2A2 distribution across gene bodies (Supplementary Figure S5). Thus, mH2A2 gene-body occupancy does not uniformly repress transcription; the active subset converges on specific biosynthetic programs.

### 2.3. Classification of mH2A2 Domains by Cell-Cycle Deposition Dynamics

Although mH2A1 is known to reload onto chromatin during mitosis [15,17], whether mH2A2 follows similar cell-cycle dynamics has remained unclear. We therefore compared the deposition pattern of both variants between G1/S and G2/M. To systematically define mH2A2 dynamics, we classified mH2A variant-enriched domains into five categories based on their relative enrichment levels across the two phases. Invariant domains showed no significant change between G1/S and G2/M. G1/S-higher and G2/M-higher domains displayed elevated deposition in one phase but remained detectable in the other (Fisher’s exact test *p*-value < 0.05), whereas G1/S-unique and G2/M-unique domains lacked detectable signal entirely in the other phase.

**Figure 3:**
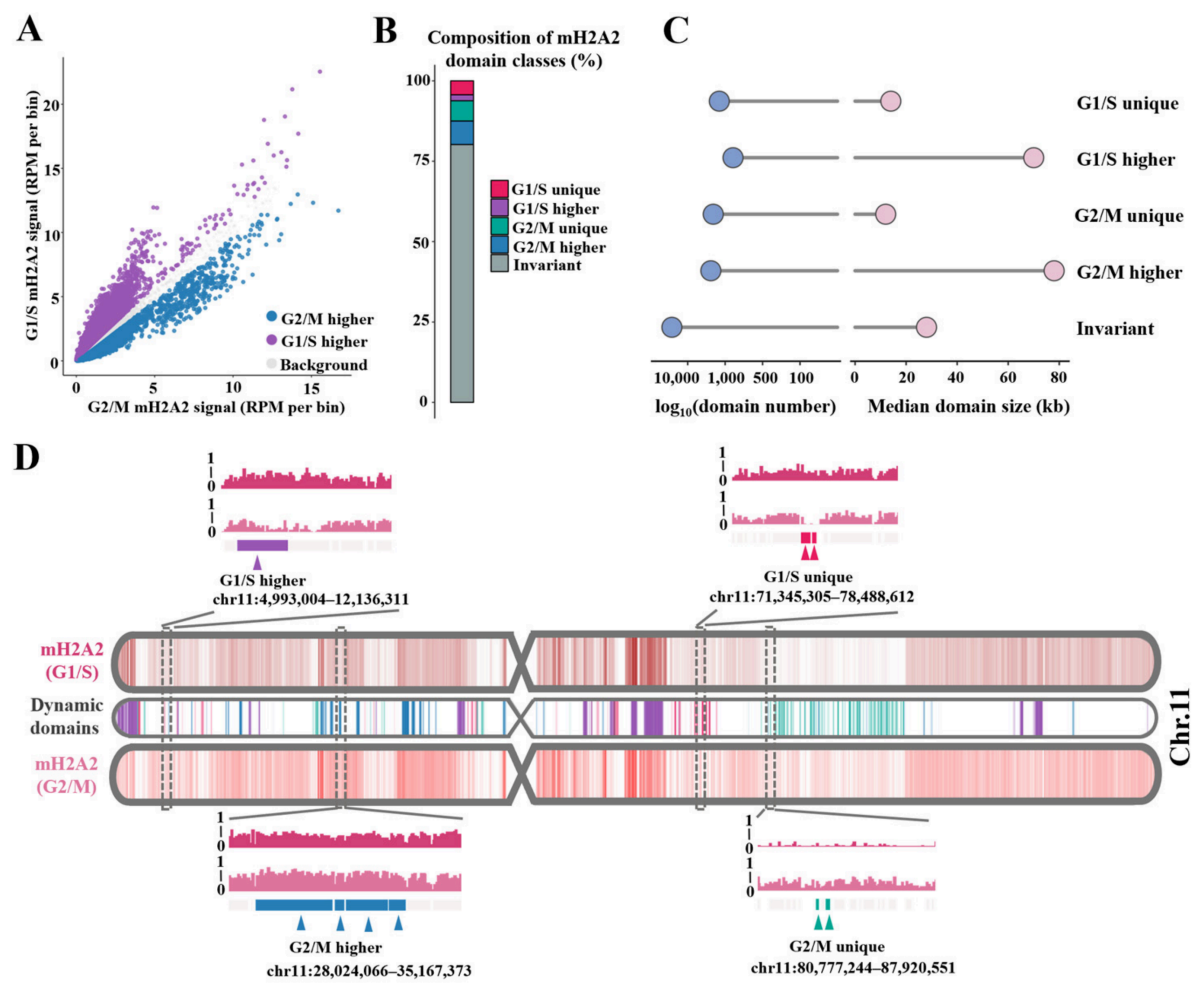
Classification of mH2A2 domains by cell-cycle deposition dynamics. (**A**) Pairwise comparison of mH2A2 signal (Read Per Million, RPM) between the G1/S and G2/M. (**B**) Proportions of the five mH2A2 domain classes. (**C**) Domain number (log10 scale, **left**) and median sizes (kb, **right**) of each domain class. (**D**) Chromosome 11 heatmaps and representative IGV tracks of dynamic mH2A2 domains (G1/S-unique, red; G1/S-higher, purple; G2/M-unique, green; G2/M-higher, blue).

Among all mH2A2 domains, 80.2% were invariant, 1.9% were G1/S-higher, 4.3% were G1/S-unique, 7.3% were G2/M-higher, and 6.3% were G2/M-unique (Figure 3B–D). Both G1/S-unique and G2/M-unique domains were relatively compact, with an average length of approximately 10 kb. In contrast, G1/S-higher and G2/M-higher domains extended over substantially larger regions (Figure 3C). Notably, although a subset of mH2A2 domains were lost at G2/M, the total genomic coverage of mH2A2 modestly increases from G1/S (69.7%) to G2/M (71.3%), driven by a larger fraction of domains that are gained or elevated at G2/M. Thus, the overall landscape is shaped by two concurrent changes: focal loss at certain regions, combined with a globally stable and slightly expanding mH2A2 landscape at G2/M.

To determine whether the cell-cycle-dependent changes in mH2A2 enrichment occur preferentially within co-occupied or variant-specific regions, we intersected each dynamic class with the co-localized, mH2A1-specific, and mH2A2-specific regions defined at G1/S (Figure 1B). The G1/S-unique, G1/S-higher, and invariant mH2A2 domains were predominantly co-occupied by both variants (76.89%, 79.02%, and 80.91%, respectively), with only a minor fraction occupied solely by mH2A2. In contrast, G2/M-unique mH2A2 domains predominantly overlapped with G1/S mH2A1-specific domains (82.6%), with the remainder located in regions uncovered by either variant during G1/S. In addition, G2/M-higher domains were mostly co-occupied by both variants (70.61%).

### 2.4. G1/S-Unique mH2A2 Domains Mark Cell-Cycle Regulatory Elements and Are Displaced at G2/M

**Figure 4:**
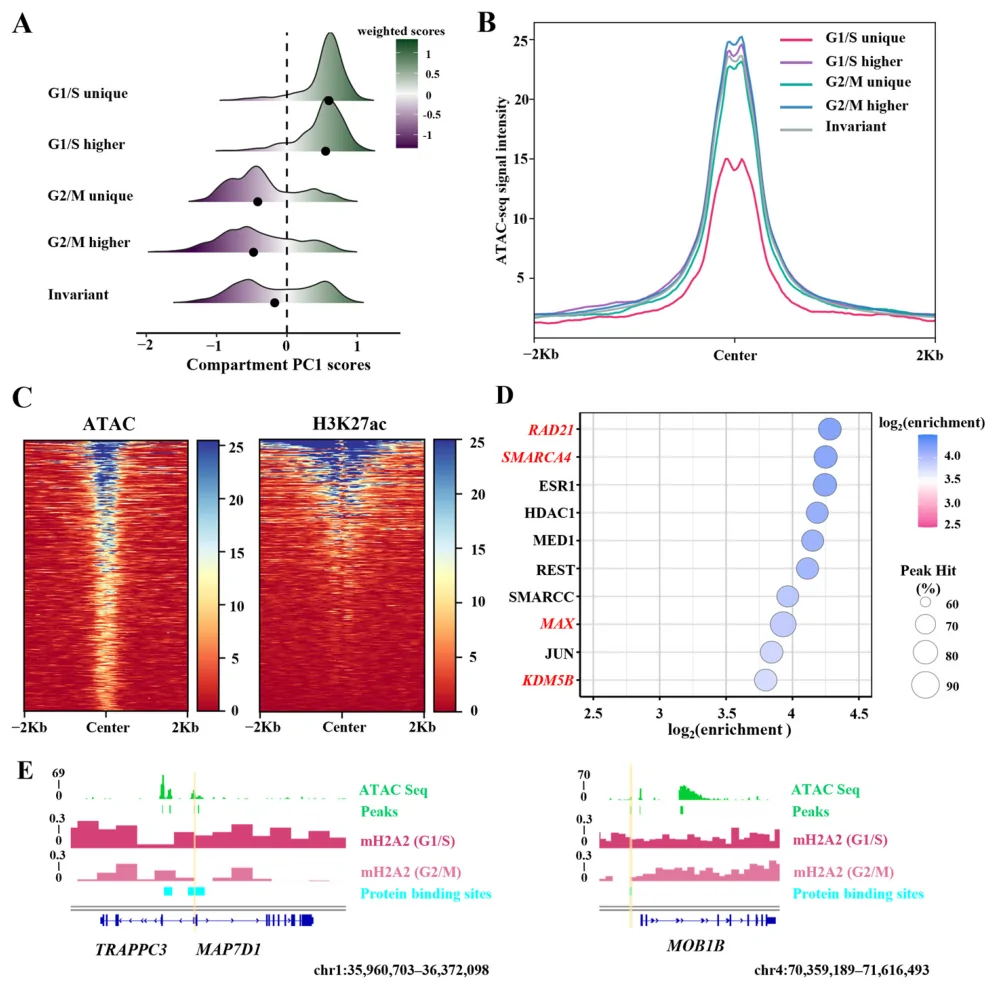
Chromatin features of dynamic mH2A2 domain classes. (**A**) Weighted Hi-C PC1 scores across domain classes (A compartment, score > 0; B compartment, score < 0; dots, medians). The dashed line in subfigure (A) indicates a PC1 score of 0. (**B**) ATAC-seq signal intensity within ±2 kb of accessible peaks in mH2A2 domains. (**C**) ATAC-seq signals and H3K27ac ChIP-seq heatmaps across PBSs in G1/S-unique domains. (**D**) Enrichment of PBSs within G1/S-unique domains. The circle size dictates the hit frequency, and the color scale reflects the log-fold enrichment relative to G2/M-higher domains. Chromatin-associated regulatory factors are italic and highlighted. (**E**) IGV snapshots of accessible peaks near promoters of cell-cycle genes in G1/S-unique domains. The yellow solid lines indicate PBSs with ATAC-seq peak signal.

To determine whether the mH2A2 domain classes are associated with different chromatin organizations, we first examined their distribution across active and repressive compartments identified by Hi-C datasets [22,23]. Among the five classes, G1/S-unique domains showed the most distinctive profile. They were strongly enriched in the active A compartment, whereas G2/M-unique and G2/M-higher domains were preferentially localized to the repressive B compartment (Figure 4A). Invariant domains, by contrast, were distributed across both compartments without apparent preference. Because mH2A2 has been reported to reduce enhancer accessibility, we next examined ATAC-seq signals across all five domain classes [24]. Because these ATAC-seq data were generated from asynchronous Huh-7 cells, they reflect an averaged accessibility state and do not directly distinguish cell-cycle stages. The lower accessibility at G1/S-unique domains should therefore be regarded as an association observed in the available datasets, and whether this reflects cell-cycle-dependent accessibility changes remains to be tested with synchronized data. Nearly all the accessible regions overlap with protein-binding sites (PBSs) annotated by ChIP-seq datasets [25], and these PBSs within G1/S-unique domains showed the lowest chromatin accessibility compared with other genomic regions (Figure 4B). Notably, 36% of these ATAC peak-associated PBSs were accompanied by H3K27ac signals, suggesting that a subset of these low-accessibility PBSs may correspond to putative *cis*-regulatory elements in Huh-7 cells (Figure 4C) [24,26]. These findings raise the possibility that mH2A2 may restrain the activity of regulatory elements during G1/S and that its displacement at G2/M may release this constraint; however, this remains an interpretation based on averaged accessibility data rather than a demonstrated outcome.

To investigate the regulatory potential of these low-accessibility sites, we further analyzed PBS enrichment within G1/S-unique domains [25]. Several cell-cycle-associated regulators, including the cohesin component RAD21, the chromatin remodeler SMARCA4, and histone demethylase KDM5B, were enriched at these sites, together with transcription factors such as MAX (Figure 4D) [27,28,29,30]. This enrichment is consistent with the notion that many G1/S-unique domains overlap regulatory elements that may participate in cell-cycle control. Since these regulatory elements may act on genes within the same chromatin compartment, we next searched for cell-cycle-associated genes located in the same A compartments as G1/S-unique domains. Our analysis identified 57 cell-cycle-associated genes within the same A compartment with these *cis*-regulatory elements covered by G1/S-unique mH2A2 domains [22,23]. These genes therefore represent candidate targets that share the same A compartment with the G1/S-unique domains; however, this co-localization does not by itself establish a direct regulatory relationship. Notably, a subset of these binding sites mapped to near the promoters of key cell-cycle regulator genes, including *MAP7D1*, which encodes a microtubule-associated protein required for mitotic spindle assembly, and *MOB1B*, a component of the mitotic exit and cytokinesis machinery (Figure 4E) [31,32]. Together, these results indicate that mH2A2 is selectively displaced from a defined subset of regulatory elements adjacent to cell-cycle genes during G2/M. This association, however, does not establish the temporal sequence or functional consequence implied by the regulatory model.

## 3. Discussion

Because mH2A1 and mH2A2 share a structurally related macrodomain and were initially identified in repressive chromatin regions, mH2A2 was long viewed as a redundant homolog of mH2A1. However, recent studies have revealed that mH2A2 has functions distinct from those of mH2A1 [3,33]. Our genome-wide comparison extends this view to the cell-cycle progression. Whereas mH2A1 undergoes broad redistribution during cell-cycle progression, mH2A2 remains largely stable, with only a subset of domains changing between G1/S and G2/M states. In addition, mH2A2 occupancy across gene bodies is not uniformly associated with transcriptional silencing. Together, these findings indicate that the two mH2A variants follow distinct transcriptional regulatory principles.

Their divergent behaviors may partly reflect structural differences between the variants. Similarly, mH2A1 is expressed as two splice isoforms, mH2A1.1 and mH2A1.2. Although both contain a macrodomain, only mH2A1.1 has a functional ADP-ribose-binding pocket. Crystallographic studies have shown that this pocket is formed by a deep hydrophobic cleft within the macrodomain [34]. This structural feature enables mH2A1.1 to participate in PARP1-dependent chromatin remodeling and may contribute to its broad redistribution across the cell cycle. By contrast, mH2A2 lacks the key coordinating residues required to form this cleft and is therefore unlikely to engage the same PARP1-dependent pathway [10]. Notably, mH2A1.2 shares this structural limitation with mH2A2, because alternative splicing replaces the exon encoding the pocket region and collapses the binding cleft [33]. Whether mH2A1.2 and mH2A2 differ in deposition kinetics or regulatory function remains an important question for future study.

G1/S-unique mH2A2 domains coincide with a less accessible chromatin state at putative cell-cycle regulatory elements and show reduced occupancy at G2/M, suggesting a phase-associated redistribution of occupancy rather than uniform genome-wide change. This model is consistent with previous observations that mH2A2 overexpression inhibits cell-cycle progression [35]. Notably, cell-cycle RNA-seq analysis showed that several regulatory proteins enriched at G1/S-unique domains, including RAD21, MAX, and KDM5B, were stably expressed from G1/S to G2/M [36]. Whether these factors bind these sites constitutively or are subject to cell-cycle-dependent exchange remains an open question, as cell-cycle-resolved ChIP-seq data for these proteins are not yet available. A broader limitation of the present study is that public repositories currently lack ATAC-seq and RNA-seq datasets from synchronized Huh-7 cells at corresponding cell-cycle stages. Consequently, whether mH2A2 displacement leads to increased accessibility and transcriptional activation of neighboring cell-cycle genes at G2/M remains an open question that will require phase-resolved ATAC-seq, RNA-seq, and ultimately experiments that directly modulate mH2A2 levels at defined cell-cycle stages.

These observations suggest that mH2A2 does not uniformly repress transcription like mH2A1 but instead exerts variable effects depending on the local chromatin environment, particularly at potential *cis*-regulatory elements. For G2/M-higher and G2/M-unique domains, mH2A2 deposition is gained or maintained at G2/M, and one possibility is that this increased occupancy contributes to reduced accessibility at these regions during mitosis. However, this interpretation also highlights an important complexity. Within invariant and G1/S-higher domains, mH2A2 is present during most of the cell cycle, yet many regulatory elements in these regions may remain active or accessible despite mH2A2 occupancy. Thus, mH2A2 occupancy alone is unlikely to uniformly predict accessibility or regulatory output across all domain classes. Instead, the effect of mH2A2 may depend on local chromatin context, including pre-existing accessibility, histone modification, PBS composition, and higher-order chromatin organization.

Neighboring genes associated with these domains further support a potential link to mitotic regulation. For instance, loss of *MAP7D1* function causes mitotic spindle defects [30], and depletion of *MOB1B*, a component of the mitotic exit machinery, disrupts mitotic progression [31]. Although these examples do not establish direct target-gene regulation, they indicate that G1/S-unique mH2A2 domains are associated with loci relevant to mitotic control. Such phase-dependent chromatin regulation is not without precedent. For example, live-cell imaging has shown that the H3K9 methyltransferase G9a is required for proper cell-cycle dynamics, with G9a loss causing a prolonged G2/M transition [37]. Similarly, cohesin is loaded and released in a cell-cycle-dependent manner, and its subunit RAD21 was enriched at the G1/S-unique mH2A2 domains identified in our study [38,39]. These examples place mH2A2 dynamics within a broader framework of phase-associated chromatin regulation rather than suggesting a direct mechanistic equivalence.

A further consideration concerns the use of A/B compartment annotations derived from HepG2 rather than Huh-7 cells [22,23]. Although A/B compartment organization is generally more conserved than local regulatory interactions, it is not identical across hepatocellular carcinoma cell lines. Consequently, the preferential localization of G1/S-unique domains within the active A compartment should be regarded as an approximation based on a reference chromatin architecture rather than a direct measurement of Huh-7 chromatin organization. This limitation means that our compartment-based interpretations remain provisional until they can be validated with matched Huh-7 Hi-C data.

The potential relevance of this mechanism may extend to cancer. mH2A2 expression is frequently reduced in aggressive tumors, including hepatocellular carcinoma, melanoma, and colorectal cancer, where its loss is associated with increased cellular plasticity and poor prognosis [39,40]. Our findings raise the possibility that cancer-associated mH2A2 loss may disrupt phase-specific regulation of cell-cycle genes programs, thereby contributing to aberrant cell-cycle transcriptional programs and uncontrolled proliferation. Directly testing this link by manipulating mH2A2 levels in cancer models and profiling chromatin accessibility by ATAC-seq and gene expression by RNA-seq across defined cell-cycle stages would help establish the relevance of mH2A2 dynamics to tumor biology [39,40,41].

In summary, our findings show that mH2A2 follows a cell-cycle-associated program distinct from that of mH2A1. Rather than undergoing broad redistribution, mH2A2 remains globally stable but is focally displaced at certain regions between G1/S and G2/M. These findings refine the classical view of macroH2A variants as static repressive chromatin components and suggest that mH2A2 combines global stability with focal, phase-associated occupancy changes at selected genomic loci.

## 4. Materials and Methods

### 4.1. Cell Cultures and Synchronization

The human hepatocellular carcinoma cell line Huh-7 was obtained from iCell Bioscience Inc. ((CL-0120, iCell Bioscience Inc., Shanghai, China). Huh7 cells were cultured in Dulbecco’s Modified Eagle medium (11320033; Gibco, Carlsbad, CA, USA) supplemented with 10% fetal bovine serum (A5209402; Gibco, Carlsbad, CA, USA) and 1% penicillin–streptomycin antibiotics (15140122; Gibco, Carlsbad, CA, USA) in an atmosphere of 95% humidified air and 5% CO2 at 37 °C.

For G1/S state synchronization, cells were first subjected to serum starvation in DMEM containing 0.5% FBS and 1% Pen/Strep for 2 days. Cells were then released into fresh complete medium supplemented with 1% Aphidicolin (ab142400, Abcam, Cambridge, MA, USA) for 18 h before harvest. For mitotic synchronization, cells were cultured to approximately 80% confluence and treated with 100 ng/mL colcemid (234109-M, Millipore, Billerica, MA, USA) for 12 h. Mitotic cells were then collected by mechanical shake-off and used for subsequent mitotic chromosome purification. Synchronization efficiency was evaluated by propidium iodide (F10797, Invitrogen, Carlsbad, CA, USA) staining followed by flow cytometry; the flow cytometry results were analyzed using FlowJo v10.8.1 (FlowJo LLC, Ashland, OR, USA).

### 4.2. Mono-Nucleosome Preparation

Mono-nucleosome preparation was adapted from previously described native ChIP procedures with modifications [42]. For mitotic samples, synchronized mitotic cells were collected by mechanical shake-off after colcemid treatment and pelleted by centrifugation at 180× *g* for 5 min. The cell pellet was resuspended in hypotonic buffer with 75 mM KCl and incubated for 30 min at 37 °C to promote cell swelling. After incubation and centrifuging at 600× *g* for 5 min at 4 °C, the pellet was resuspended in polyamine buffer to preserve mitotic chromosome integrity. The cell suspension was homogenized on ice using a 7 mL Dounce homogenizer (D9063-1SET, Sigma-Aldrich, St. Louis, MO, USA) with intermittent pauses. Large debris was removed by centrifugation at 190× *g* for 5 min at 4 °C, and the remaining supernatant was sequentially filtered through 10 μm (NY1002500, Millipore, Billerica, MA, USA) and 5 μm (AN5004700, Millipore, Billerica, MA, USA) membranes. Purified mitotic chromosomes were then collected by centrifugation at 1750× *g* for 10 min at 4 °C. The chromosome pellet was resuspended in MNase (micrococcal nuclease) buffer (15 mM Tris-HCl pH 7.5, 0.5 mM EGTA, 2 mM EDTA, 80 mM KCl, 20 mM NaCl, 0.5 mM spermidine, 0.2 mM spermine, 35 mM CaCl_2_, 35 mM Tris-HCl pH 8.0, 10 μg/mL BSA, 1 mM PMSF) and protease inhibitor cocktail(04693116001, Roche Diagnostics GmbH, Mannheim, Germany). Mitotic chromosomes were digested with MNase (M0247S, NEB, Ipswich, MA, USA) with 3000 gel units/mL at 4 °C overnight, and the reaction was terminated by adding EDTA to a final concentration of 20 mM. The digested samples were centrifuged at 10,000 × rpm for 7 min at 4 °C, and the supernatant containing mitotic mono-nucleosomes was collected for native ChIP.

For G1/S samples, synchronized cells were harvested by trypsin (25200072; Gibco, Carlsbad, CA, USA) digestion at 37 °C for 2 min and washed twice with ice-cold phosphate-buffered saline (PBS). Cells were then resuspended in MNase buffer supplemented with 0.5% NP-40 (56741-50ML-F, Sigma-Aldrich, St. Louis, MO, USA) to permeabilize the membrane and release chromatin. G1/S chromatin was digested with MNase at 2000 gel units/mL under equivalent conditions at 4 °C overnight. The digestion products were centrifuged at 10,000 × rpm for 7 min at 4 °C, and the supernatant containing G1/S mono-nucleosomes was collected for native ChIP.

### 4.3. nChIP-Seq and Sequencing Library Preparation

nChIP was performed using mono-nucleosomes derived from either G1/S-phase chromatin or purified mitotic chromosomes. For each ChIP reaction, the corresponding antibody was pre-incubated with Protein A/G magnetic beads (16-663, Millipore, Billerica, MA, USA) for 1 h at 4 °C with rotation according to the manufacturer’s instructions. The antibody-conjugated beads were then incubated with mono-nucleosome preparations overnight at 4 °C with continuous rotation.

The following antibodies were used for native ChIP: rabbit recombinant monoclonal anti-mH2A1 antibody (ab183041, Abcam, Cambridge, MA, USA) and mouse monoclonal anti-mH2A2 ChIP antibody (sc-377452 X, Santa Cruz Biotechnology, Dallas, TX, USA). After immunoprecipitation, beads were washed, and bound nucleosomes were eluted according to the bead manufacturer’s recommended protocol. Input mono-nucleosomes and ChIP-enriched nucleosomes were treated with 100 μg/mL RNase A (AM2271, Invitrogen, Carlsbad, CA, USA) to remove RNA contamination, followed by overnight digestion with 1% SDS and 200 μg/mL Proteinase K (4333793, Invitrogen, Carlsbad, CA, USA) at 56 °C. DNA was purified by phenol-chloroform extraction and ethanol precipitation.

Sequencing libraries were prepared using approximately 1 ng of purified DNA per sample with the NEBNext^®^ Multiplex Oligos (E7500S, NEB, Ipswich, MA, USA). Libraries were sequenced on the MGI^®^ DNBSEQ-T7 platform (DNBSEQ-T7, MGI Tech Co., Ltd., Shenzhen, China).

### 4.4. ChIP-Seq Data Processing

Raw paired-end sequencing reads from mH2A1 and mH2A2 nChIP-seq libraries were processed using the same analysis workflow. Adapter sequences and low-quality bases were removed using TrimGalore (https://github.com/FelixKrueger/TrimGalore, accessed on 16 July 2026), and reads with a quality score below Q20 were discarded. The filtered reads were aligned to the human reference genome GRCh38/hg38 using Bowtie2 (v2.3.4.3) [43]. Reads mapped to unmapped or unplaced chromosome segments, mitochondrial DNA, or multiple genomic locations were removed using SAMtools (v1.9) [44]. The remaining mapped reads were converted to BAM format and uniquely mapped non-duplicated reads were retained for downstream analysis.

Genome-wide signal tracks were generated from processed BAM files using bamCoverage from deepTools and exported as BigWig files for visualization [45]. The reproducibility between biological replicates was assessed using multiBamSummary from deepTools (v3.3.0) with 2 kb genomic bins, followed by Pearson correlation analysis and visualization using plotCorrelation from deepTools [45]. Highly correlated biological replicates were combined for subsequent domain calling and comparative analyses.

mH2A1- and mH2A2-enriched regions were identified separately for G1/S and mitotic samples using normR (v3.21), with the corresponding input nucleosome DNA used as the normalization control [46]. The entire genome was divided into consecutive 2 kb bins, and the midpoints of mapped reads were counted within each bin. For each bin, ChIP enrichment relative to input was calculated by normR [46]. Significantly enriched bins were identified using Fisher’s exact test with a threshold of *p*-value < 0.05. The enriched bins were exported as BED and BigWig files using exportR, and adjacent enriched bins were merged into mH2A-enriched regions. The resulting mH2A1 and mH2A2 domains were used for downstream analyses using BEDTools (v2.30.0) [47]. Illustrative genomic regions were visualized with Integrative Genomics Viewer (IGV) [48]. The complete lists of mH2A1- and mH2A2-enriched domains identified at G1/S and G2/M are provided as Supplementary Tables S2–S5.

To compare the relative enrichment of mH2A1 and mH2A2 within co-occupied regions, differential enrichment was statistically assessed using diffR [46], and *p*-values were adjusted for multiple testing using the Storey method to obtain q-values (FDR). Regions with a foldchange greater than 1.5 in either direction and a q-value < 0.05 were classified as showing higher enrichment of the corresponding variant. All remaining regions were classified as showing comparable enrichment of the two variants. To systematically define cell-cycle-associated regional signal changes, we performed differential analysis between matched G1/S and mitotic samples within the same 2 kb genomic bins. Differential bins were classified according to the direction of signal change between G1/S and G2/M. Using Storey’s false discovery rate correction (FDR) with a threshold of 0.05, we identified 170,949 G1/S higher bins, 352,685 G2/M higher bins, and 1,020,512 bins showing no significant difference across cell-cycle phases. These differential and invariant bins were further merged or intersected with mH2A domains to define phase-associated domain classes.

### 4.5. RNA Data Analysis

Raw RNA-seq data of Huh-7 cells were obtained from GEO (GSE190076) [49]. Adapter trimming and quality filtering were performed using TrimGalore, and reads with Q < 20 were removed. The filtered reads were aligned to the human reference genome GRCh38/hg38 using HISAT2 (2.2.1) [50]. Unmapped and multi-mapped reads were excluded, and uniquely mapped reads were retained.

A count table was generated using featureCounts (v2.0.1) with the corresponding human gene annotation [51]. Expression levels were normalized as RPKM (Reads Per Kilobase per Million mapped reads). RNA-seq coverage tracks were generated using deepTools for visualization [45].

### 4.6. Public Dataset Citation

The processed ATAC-seq and H3K27ac ChIP-seq BigWig signal file and annotated peak BED files of Huh-7 cells were downloaded from GEO (GSE184797) [24].

Non-redundant protein-binding site annotations were downloaded from the ReMap2022 database and used to annotate the ATAC-seq peak located within mH2A2 domain categories [25].

A/B compartment information was obtained from PC1-like compartment scores derived from Hi-C data of HepG2 cells (4DN Data Portal dataset 4DNESC2DEQIJ). Because HepG2 shares a related hepatocellular carcinoma origin and broadly comparable large-scale chromatin features with Huh-7, and because A/B compartment organization represents relatively stable large-scale chromatin segregation that is generally more conserved than locus-specific regulatory features, these annotations were used as a broad reference framework for classifying active (A) versus repressive (B) compartments. We note that these compartment annotations provide a reference rather than a direct measurement of Huh-7-specific 3D genome organization. The compartment bins were intersected with mH2A2 domains, and the compartment score in each domain was calculated as the sum of PC1-like scores weighted by intersected length and normalized to the total length of each domain.

## 5. Conclusions

This study reveals that mH2A2 follows a cell-cycle program fundamentally distinct from that of mH2A1. Rather than undergoing broad redistribution across the genome, mH2A2-enriched domains remain largely invariant, with only a small fraction displaying focal, phase-specific displacement. These dynamic sites, particularly the G1/S-unique domains, preferentially locate within the active chromatin compartment and coincide with reduced accessibility at binding sites for cell-cycle regulators. Their enrichment near genes involved in mitotic progression suggests a model in which mH2A2 temporarily restrains regulatory elements during G1/S and is selectively released upon G2/M to facilitate phase-specific gene activation. This proposed mode of regulation expands the classical view of macroH2A variants beyond that of static transcriptional repressors and highlights their potential to function as temporally responsive components of chromatin control. Further studies combining targeted perturbation of mH2A2 occupancy with higher-resolution epigenomic profiling in both normal and disease settings will be essential to fully define the scope and significance of this regulatory mechanism.

## Data Availability

The ChIP-seq data presented in this study have been deposited in the Gene Expression Omnibus (GEO) under accession number GSE337818.

## Supplementary Materials

The following supporting information can be downloaded at https://www.mdpi.com/article/10.3390/ijms27177708/s1.

## Abbreviations

The following abbreviations are used in this manuscript:

ATAC-seq	Assay for Transposase-Accessible Chromatin using Sequencing
CUT&RUN	Cleavage Under Targets and Release Using Nuclease
CUT&Tag	Cleavage Under Targets and Tagmentation
GO	Gene Ontology
Hi-C	High-throughput Chromosome Conformation Capture
Huh-7	Human Hepatocellular Carcinoma Cell Line 7
mH2A1	MacroH2A1
mH2A2	MacroH2A2
nChIP-seq	Native Chromatin Immunoprecipitation Sequencing
PBS	Protein Binding Site
RPKM	Reads Per Kilobase per Million Mapped Reads
RNA-seq	RNA Sequencing
