# Supplementary material for: MacroH2A2-Enriched Domains Are Largely Stable Across the Cell Cycle but Focally Displaced at Mitotic Regulatory Elements
Source: Int J Mol Sci. 2026 Aug 28;27(17):7708. doi: 10.3390/ijms27177708 (PMC13566895; doi:10.3390/ijms27177708)
Supplement: Supplementary file 1 [file ijms-27-07708-s001.zip › ijms-supplementary.pdf]

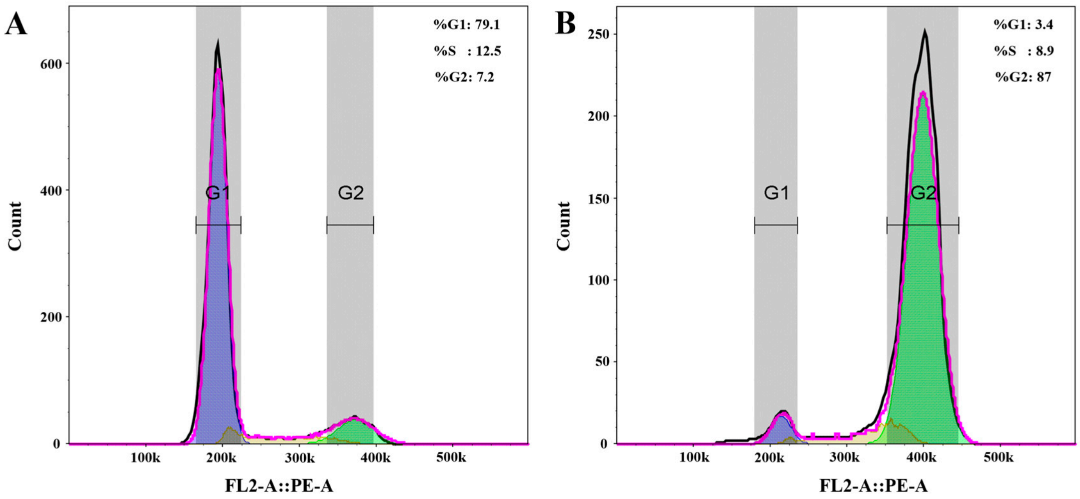

Figure S1. Validation of cell-cycle synchronization in G1/S and G2/M states.

Flow cytometry analysis showing the cell-cycle distribution of synchronized cells used for nChIP-seq. (**A**) G1/S synchronization, (**B**) G2/M synchronization.

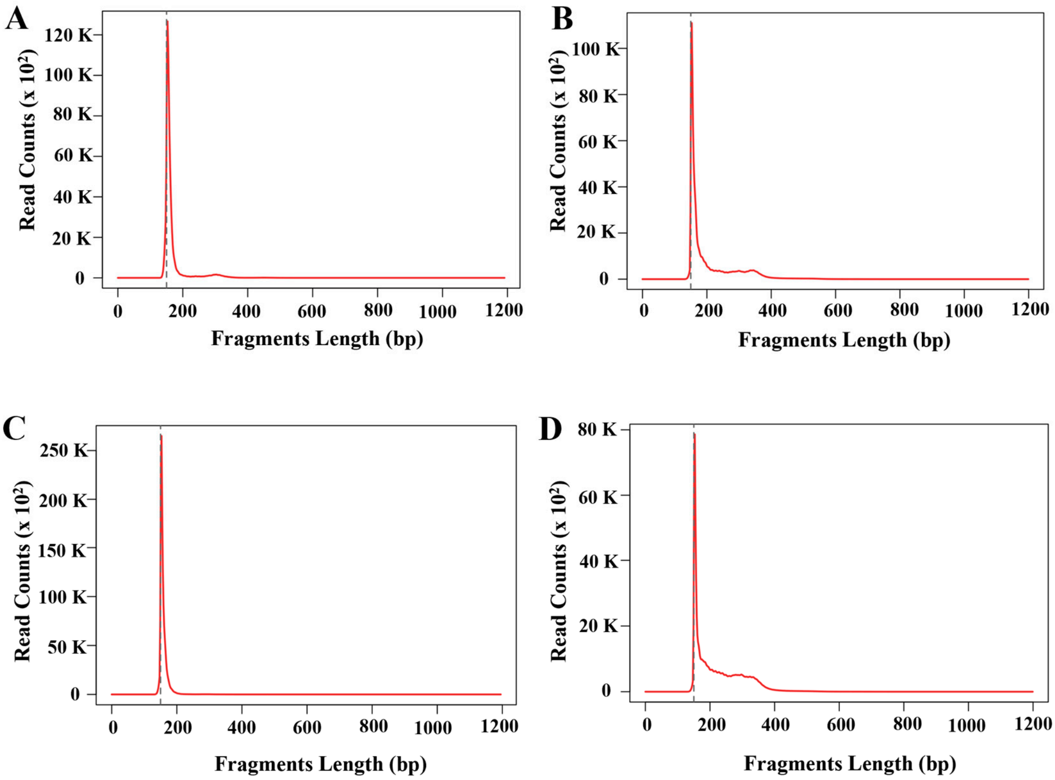

Figure S2. Quality assessment of nChIP-seq libraries.

Fragment length distribution of nChIP-seq libraries showing the predominant enrichment of mono-nucleosome-sized fragments. The vertical dashed line indicates 150 bp. (A) mH2A1 in G1/S states, (B) mH2A1 in G2/M, (C) mH2A2 in G1/S, (D) mH2A2 in G2/M.

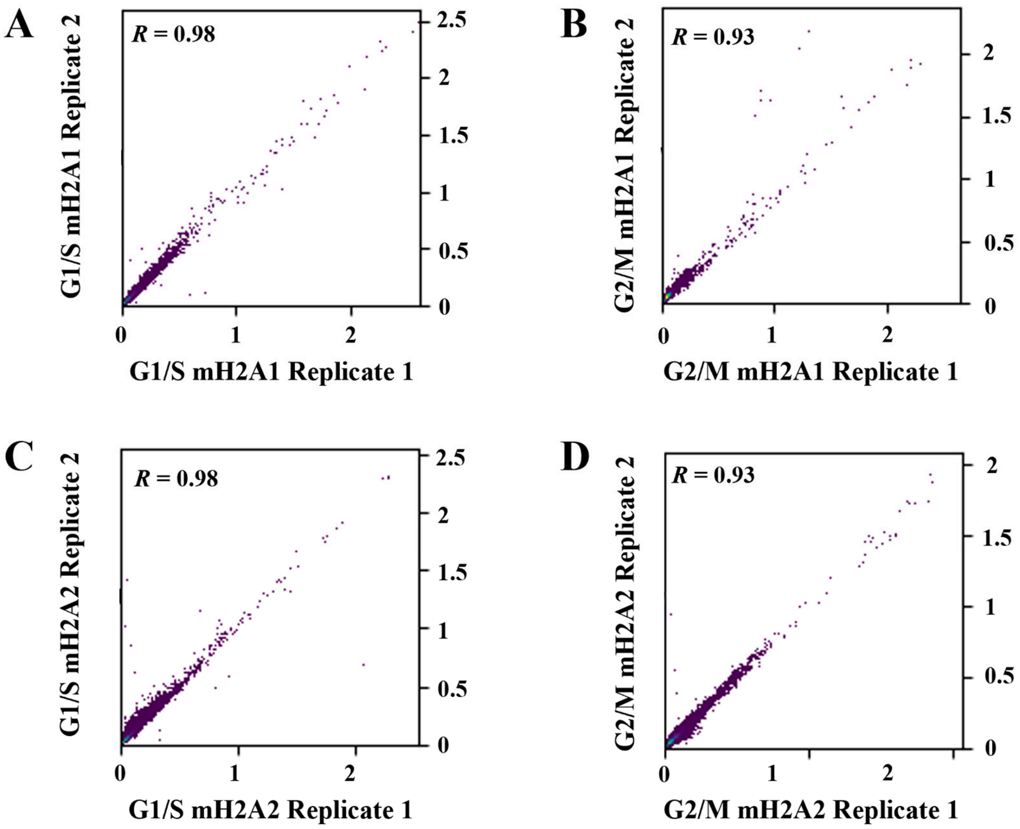

Figure S3. Correlation of biological replicates of mH2As nChIP-seq library

The replicates of the nChIP-seq data are highly consistent for both G1/S and G2/M cells. Shown are the values of the Pearson correlation coefficient (R).

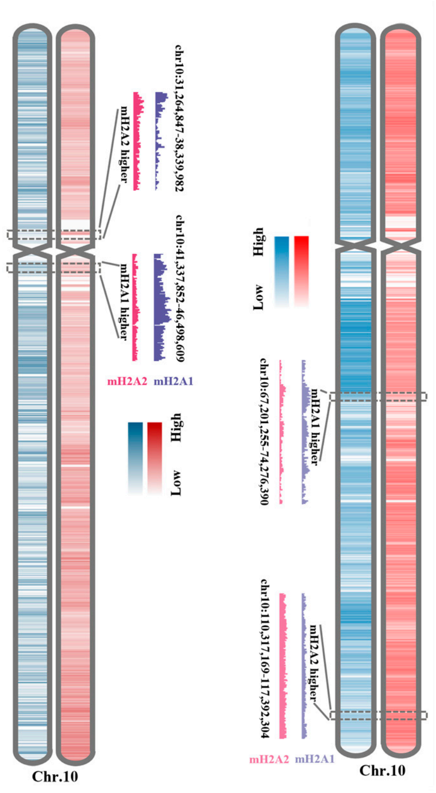

Figure S4. Illustrative chromosome-wide heatmaps of mH2A1 and mH2A2 enrichment on Chr. 10.

Two representative genomic regions are shown for the G1/S (left) and G2/M (right) states, exemplifying mH2A1-higher, and mH2A2-higher regions, respectively.

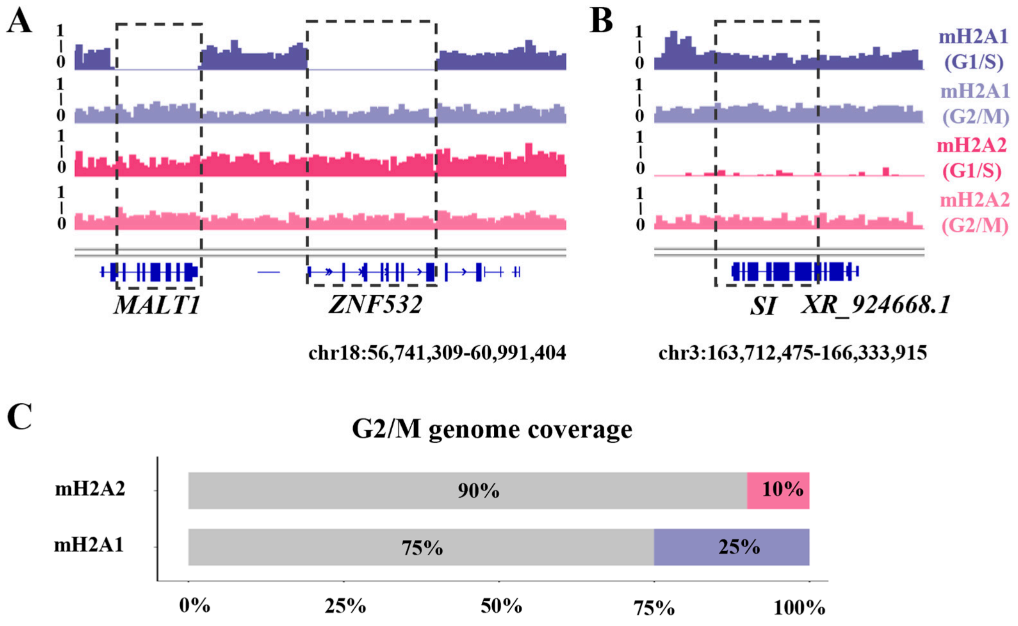

Figure S5. Distinct cell-cycle dependent deposition patterns of mH2A1 and mH2A2.

The distinct cell-cycle dependent deposition pattern of mH2A1 (**A**) and mH2A2 (**B**) at the same genomic regions. (**C**) Comparison of G2/M-increased coverage between mH2A1 and mH2A2.

Table S1. Summary of the sequencing and mapping quality of nChIP libraries

	G1/S mH2A1 Control	G1/S mH2A1 Replicate 1	G1/S mH2A1 Replicate 2	G2/M mH2A1 Control	G2/M mH2A1 Replicate 1	G2/M mH2A1 Replicate 2	G1/S mH2A2 Control	G1/S mH2A2 Replicate 1	G1/S mH2A2 Replicate 2	G2/M mH2A2 Control	G2/M mHA2 Replicate 1	G2/M mH2A2 Replicate 2
Raw Reads	325,780,294	359,464,478	431,560,830	236,063,680	295,657,906	304,512,954	280,951,406	385,125,096	474,881,920	350,830,924	236,533,090	238,421,610
Mapped Reads	254,477,650	241,291,305	345,947,063	222,971,929	284,867,874	287,974,781	185,670,690	350,815,705	438,506,745	329,558,352	228,316,937	228,319,393
Uniquely Mapped Reads	155,212,336	94,498,657	153,952,793	185,672,916	238,137,114	236,258,474	86,299,536	218,085,940	277,729,798	260,340,025	184,416,236	182,811,240
Duplicate Rate	4.21%	6.69%	8.50%	2.62%	20.40%	18.53%	4.38%	16.87%	20.16%	4.12%	16.81%	17.82%

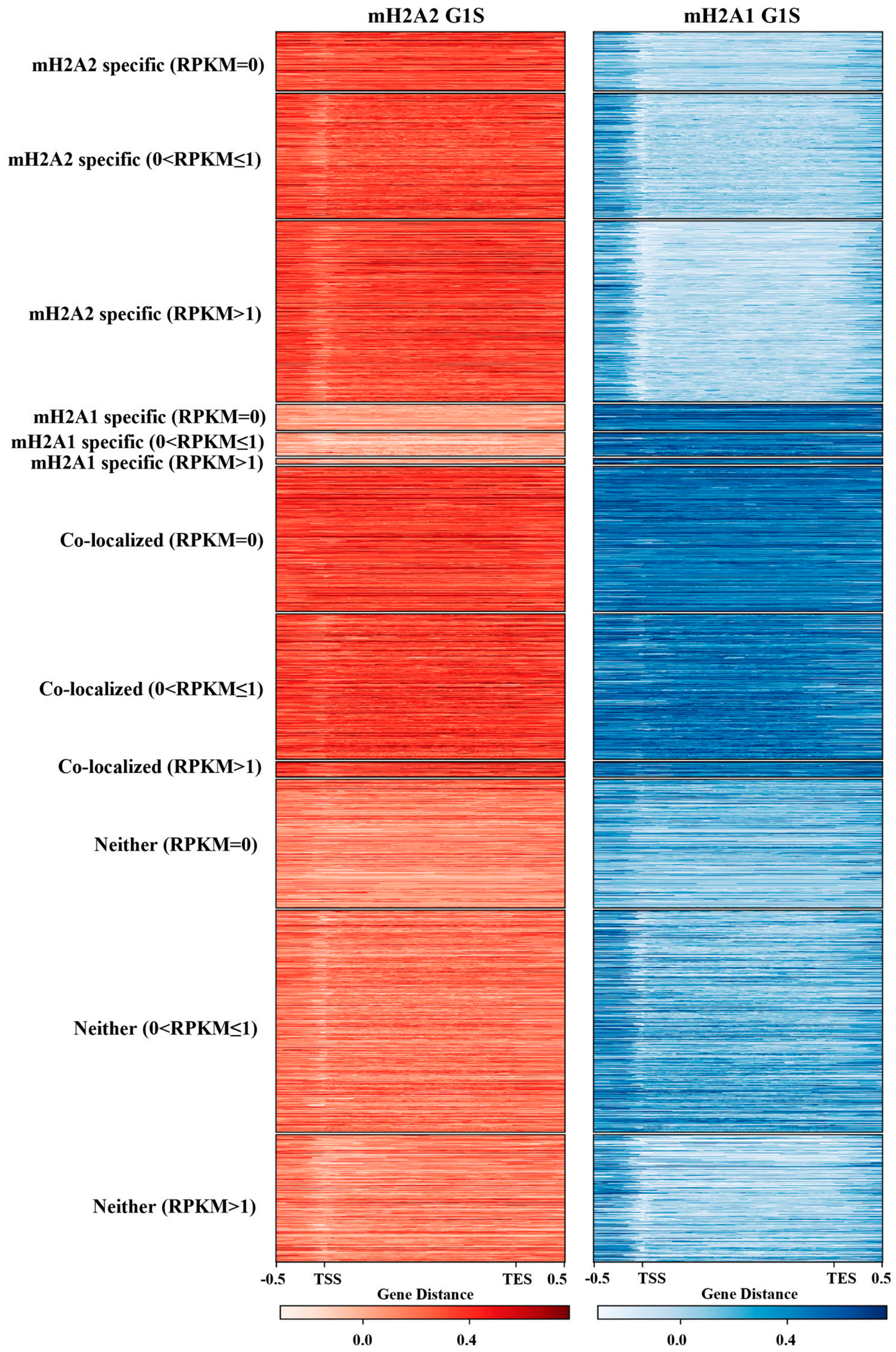

Figure S6. Heatmap representation of mH2A1 and mH2A2 enrichment across gene bodies in G1/S-phase cells.

ChIP-seq signal intensities for mH2A2 (left, red scale) and mH2A1 (right, blue scale) are plotted across normalized gene bodies (from transcription start site [TSS] to transcription end site [TES]). Genes were classified into four occupancy categories based on mH2A1 and mH2A2 enrichment patterns: mH2A2-specific, mH2A1-specific, Co-localized, and Neither. Within each category, genes were further stratified by expression level (RPKM = 0, 0 < RPKM ≤ 1, and RPKM > 1), and sorted from highest to lowest expression within each stratum.

Supplemental methods

Mitotic chromosome isolation and purification

Mitotic cells were collected by mechanical shake-off and pelleted by centrifugation at 180 × g for 5 min. To remove residual PBS and ensure efficient hypotonic swelling, the cell pellet was first washed once with 10 mL 75 mM KCl at room temperature. Cells were then collected by centrifugation at 200 × g for 5 min, and the supernatant was carefully removed. For hypotonic treatment, the cell pellet was first gently resuspended in 1 mL hypotonic buffer (75 mM KCl) to fully disperse the cells. The suspension was then brought to a final volume of 10 mL with same hypotonic buffer (75 mM KCl) and gently mixed by pipetting. Mitotic cells were incubated in a molecular hybridization at 30 °C with moderate rotation for 30 min to promote cell swelling. After hypotonic treatment, cells were collected by centrifugation at 600 × g for 5 min at 4 °C.

The swollen cell pellet was resuspended in 1 mL ice-cold polyamine buffer (15 mM Tris-HCl pH 7.5, 0.5 mM EGTA, 2 mM EDTA, 80 mM KCl, 20 mM NaCl, 0.5 mM spermidine, 0.2 mM spermine, 0.1 mM PMSF, and 1 μg/mL digitonin) and gently dispersed by pipetting while avoiding air bubbles. An additional 4 mL of polyamine buffer was then added, and the suspension was gently mixed and kept on ice for 10 min[42].

The cell suspension was homogenized on ice using a 7 mL Dounce homogenizer (D9063-1SET, Sigma-Aldrich). Homogenization was first performed with the loose pestle for 15 strokes at a constant speed to avoid air bubbles, with a 30 s pause after every 5 strokes. The suspension was then further homogenized with the tight pestle for 12 strokes at a constant speed, with a 30 s pause after every 3 strokes. The homogenate was centrifuged at 190 × g for 5 min at 4 °C to remove large debris. The supernatant was carefully collected and slowly pushed through 10 μm and 5 μm filter membranes by horizontally positioned syringe pump to minimize mechanical shearing and preserve chromosome integrity. The filtered chromosome suspension was centrifuged at 1750 × g for 10 min at 4 °C, the pellet was gently resuspended in the appropriate downstream buffer for MNase digestion and nChIP. To assess the quality of chromosome purification, an aliquot of purified mitotic chromosomes was stained with DAPI and examined by fluorescence microscopy (Fig S5).

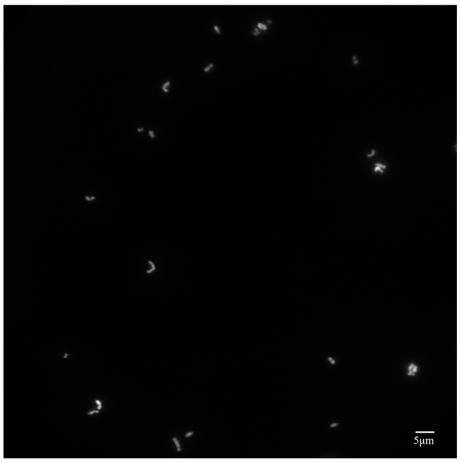

Figure S7. DAPI staining of purified mitotic chromosomes.

Representative fluorescence microscopy image of purified mitotic chromosomes stained with DAPI. The condensed chromosome morphology confirmed the successful enrichment and preservation of mitotic chromosomes after isolation and purification.

Reference

42. Meng, Y.; Yi, X.; Li, X.; Hu, C.; Wang, J.; Bai, L.; et al. The Non-Coding RNA Composition of the Mitotic Chromosome by 5′-Tag Sequencing. Nucleic Acids Res. 2016, 44, 4934–4946.
